# The distal end of porcine chromosome 6p is involved in the regulation of skatole levels in boars

**DOI:** 10.1186/1471-2156-12-35

**Published:** 2011-04-20

**Authors:** António M Ramos, Naomi Duijvesteijn, Egbert F Knol, Jan WM Merks, Henk Bovenhuis, Richard PMA Crooijmans, Martien AM Groenen, Barbara Harlizius

**Affiliations:** 1 Animal Breeding and Genomics Centre, Wageningen University, PO Box 338, 6700 AH, Wageningen, The Netherlands; 2 IPG, Institute for Pig Genetics B.V., PO Box 43, 6640 AA, Beuningen, The Netherlands

## Abstract

**Background:**

Boar taint is an unpleasant condition of pork, mainly due to the accumulation of androstenone and skatole in male pigs at onset of puberty. This condition is the cause of considerable economic losses in the pig industry and the most common practice to control it is to castrate male piglets. Because of the economic and animal welfare concerns there is interest in developing genetic markers that could be used in selection schemes to decrease the incidence of boar taint. The Porcine 60 K SNP Beadchip was used to genotype 891 pigs from a composite Duroc sire line, for which skatole levels in fat had been collected.

**Results:**

The genome-wide association study revealed that 16 SNPs (single nucleotide polymorphisms) located on the proximal region of chromosome 6 were significantly associated with skatole levels. These SNPs are grouped in three separate clusters located in the initial 6 Mb region of chromosome 6. The differences observed between the homozygote genotypes for SNPs in the three clusters were substantial, including a difference of 102.8 ng/g skatole in melted fat between the homozygotes for the ALGA0107039 marker. Single SNPs explain up to 22% of the phenotypic variance. No obvious candidate genes could be pinpointed in the region, which may be due to the need of further annotation of the pig genome.

**Conclusions:**

This study demonstrated new SNP markers significantly associated with skatole levels in the distal region of chromosome 6p. These markers defined three independent clusters in the region, which contain a low number of protein-coding genes. The considerable differences observed between the homozygous genotypes for several SNPs may be used in future selection schemes to reduce skatole levels in pigs

## Background

Boar taint is characterized as the unpleasant odour and flavour that is released from pork when it is heated. This undesirable condition in meat from a certain proportion of finishing boars is perceived as offensive by consumers and leads to economic losses through the rejection of tainted carcasses. The most common practice to prevent boar taint is castration of male piglets. However, this is an objectionable practice because of animal welfare concerns and a ban on castration is viewed as a likely measure to be adopted by the European Union [1]. In addition, castrated males have inferior productive performances when compared with un-castrated males, namely worse feed conversion and lower lean content [2]. Hence, there is considerable interest in developing a solution for boar taint that does not involve castration.

Boar taint is mainly caused by abnormally elevated levels of androstenone and/or skatole. Androstenone is a steroid produced in the testis using cholesterol as a precursor [3], and later on is metabolized in the testis or in the liver [4,5]. Skatole is a metabolite of tryptophan and is produced by intestinal bacteria in the pig gut [6]. In pigs the metabolism of skatole occurs in the liver and is performed in two different phases, an initial phase involving enzymes from the cytochrome P450 family [7] and a second phase involving a sulfation reaction by a phenol sulfotransferase [8].

To date, several studies have focused on finding genes and chromosomal regions involved in the regulation of skatole levels. A QTL for skatole was identified on pig chromosome 6 (SSC6) [9] in a Landrace pig population, using a Bayesian QTL mapping approach. Further evidence for the involvement of SSC6 on the regulation of skatole levels was provided in a study where QTL for skatole were also identified in two different SSC6 regions, using a Large White × Meishan crossbred population [10]. In addition, the same authors also discovered skatole QTL on SSC13 and SSC14, with two QTL identified on each of these chromosomes. Finally, single QTL for skatole levels were also identified on SSC7, SSC12 and SSCX, also utilizing a Large White × Meishan pig population [11].

In addition to the QTL studies mentioned previously, other authors focused on the analysis of individual genes and their possible impact on the regulation of skatole levels in the pig. In this regard, genes coding for enzymes of the cytochrome P450 family received considerable interest, due their role in the skatole metabolism. It was suggested initially that low levels of CYP2E1 in the liver may be responsible for higher levels of skatole in fat because of decreased metabolism and clearance of skatole [12,13], indications that were later on confirmed [14]. The CYP2E1 gene was subsequently mapped to the distal end of SSC14q [15], in a study where a SNP in the promoter region of this gene was also found to be significantly associated with skatole levels. Furthermore, significant associations between SNPs within the CYP2E1 and CYP21 genes and reduced skatole levels were identified, providing further support for the involvement of the cytochrome P450 enzymes in skatole regulation [16]. The participation of another member of this family, CYP2A6, has also been demonstrated. In particular, a frame shift mutation in the coding region of the CYP2A6 gene producing a non-functional enzyme was found to be associated with higher levels of skatole in fat [17]. Finally, the CYB5A gene, which is involved in the synthesis of androstenone and may activate CYP2E1 and CYP2A6 in humans [18], was also found to be associated with skatole levels in Duroc and other sire lines [19], but this effect was not observed across all pig lines analyzed.

The objectives of this work were to perform a genome wide association study with the PorcineSNP60 bead chip [20] to identify genetic markers for skatole levels in a population of Duroc pigs.

## Methods

### Animals and phenotypes

DNA samples were available for a total of 954 individuals from a composite Duroc sire line. These boars were selected from a dataset of 1663 animals based on discordant sib pairs and originated from 57 sires and 212 dams [21].

Phenotypic measurements for skatole levels were obtained using fat samples collected in all the individuals. Mean skatole levels in fat were 91.11 ng/g melted fat (± 97.48 ng/g). The phenotypic values for skatole were not normally distributed, hence they were subjected to a logarithmic transformation. The skatole levels used in the association analysis were corrected previously for several systematic environmental effects, which included hot carcass weight, age at slaughter and backfat depth at slaughter as covariates, and batch and litter as random effects. Additional details regarding phenotype collection are provided elsewhere [21]. Heritability for skatole in this dataset was estimated as 0.51 using ASReml [22] which is within the range of 0.19 and 0.55 reported in other studies [23,24].

### Genotypes

The PorcineSNP60 bead chip [20] containing 64,232 SNP markers was used to genotype all samples at ServiceXS (Leiden, The Netherlands). The genotype data were then evaluated for several quality criteria. A total of 63 animals were removed from the dataset because they contained an excessive number of SNPs displaying pedigree errors. An animal was considered to have an excessive number of pedigree errors when the percentage of correct genotypes was lower than 99%. After removal of these individuals, a total of 891 animals remained in the dataset. In total there were 113 singletons and 778 boars in discordant full sib pairs (2 or more full sibs). The average relatedness among the 891 animals with 3 generations of pedigree was 0.10 varying between 0 and 0.80.

Additional criteria were used to filter the SNP data, including for each SNP the number of pedigree errors, mapping status on build9 assembly of the pig genome, average GenCall score and minor allele frequency. SNPs containing in excess of 30 pedigree errors were removed, which resulted in the exclusion of 122 SNPs. Additionally, 12,597 SNPs that were not unambiguously mapped to build9 of the pig genome were removed. This number included SNPs that were not found on build9 and SNPs that could not be uniquely assigned to a specific porcine chromosome. Finally, SNPs that displayed an average GenCall score lower than 0.7 and/or a minor allele frequency lower than 1% were also discarded, which resulted in the removal of 7,877 markers. The GenCall score of 0.7 was chosen to improve data quality and a MAF of 1% to be able to detect rare variants. After application of all filtering criteria, a total of 43,636 SNPs remained in the dataset.

### Association analysis

The log transformed skatole values were analyzed as a quantitative trait with the QFAM module of PLINK [25]. This module implemented a within-sib-ship test that accounted for the family structure present in the dataset. An adaptive permutation procedure was used to obtain empirical p-values. This permutation procedure accounts for the family relationships between individuals, thus correcting for family structure, and its adaptive nature derives from the fact that it prioritizes the permutation procedure for the more promising SNPs, in terms of significance. In practice, SNPs that will clearly be not significant after a limited number of permutations are discarded, while permutation continues for the more promising SNPs up to 1,000,000 permutations. Score inflation introduced by familiar relations was corrected for using genomic control. To correct for the multiple testing of thousands of SNPs the package q-value, developed by Dabney and Storey [26] and implemented in R, was used. This was performed by calculating a q-value for each of the p-values previously determined, that was subsequently used to assess significance at the chromosome level. Significant associations were declared when the q-value for each marker was less than 0.05.

Linkage disequilibrium between SNPs was determined using Haploview version 4.2 [27]. The variance explained by a significant SNP was calculated using ASReml [22]. The log-transformed skatole was analysed and the model included systemic environmental effects (as described earlier), a polygenic effect and the SNP (included as a random effect).

The fraction of the phenotypic variance explained by the SNP = .

## Results and Discussion

**Figure 1:**
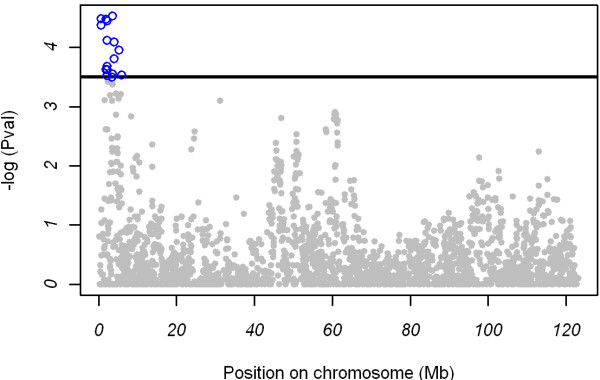
**Association between skatole levels and 2,418 SNPs located on SSC6**. On the y-axis are -log10(p-values) and on the x-axis are the physical positions of the SNPs. The significance threshold used was 3.50, which corresponds to a chromosome-wide FDR q-value of 0.05.

A total of 16 SNPs were significantly associated with skatole levels at the chromosome-wide q-value ≤ 0.05 (Table 1). These markers were all located on SSC6 in the initial 6 Mb of this chromosome (Figure 1). All the remaining SNPs located in other regions of the genome were not significantly associated with skatole.

**Table 1:** List of the SNP markers significantly associated with skatole levels in the pig population analyzed

		Allele Frequency				
					
SNP	Position	Major Allele	Minor Allele	Regression Coefficient	p-value	q-value
*Region 1*						
MARC0019446	636,696	T: 0.52	G: 0.48	0.26	0.0000323	0.02
ASGA0084674	645,690	T: 0.51	G: 0.49	0.26	0.0000420	0.02
*Region 2*						
MARC0044930	1,795,517	G: 0.77	A: 0.23	0.31	0.0000331	0.02
ASGA0096130	1,802,015	A: 0.74	C: 0.26	0.24	0.0002333	0.05
ASGA0098975	1,939,519	G: 0.76	A: 0.24	0.32	0.0000758	0.02
ALGA0107039	2,113,937	G: 0.77	T: 0.23	0.30	0.0000352	0.02
ALGA0116170	2,117,567	G: 0.76	T: 0.24	0.27	0.0002347	0.05
ALGA0115841	2,125,025	C: 0.76	T: 0.24	0.30	0.0002955	0.05
ALGA0110693	2,156,144	C: 0.73	A: 0.27	0.31	0.0002065	0.05
*Region 3*						
ALGA0115538	3,353,995	G: 0.70	A: 0.30	0.25	0.0003122	0.05
MARC0034202	3,573,079	G: 0.79	A: 0.21	0.27	0.0002793	0.05
ALGA0034369	3,596,641	A: 0.75	G: 0.25	0.31	0.0000290	0.02
MARC0067306	3,923,036	T: 0.73	C: 0.27	-0.26	0.0000801	0.03
MARC0019712	3,936,297	C: 0.79	T: 0.21	0.29	0.0001532	0.04
*Isolated Markers*						
MARC0009863	5,222,760	G: 0.51	A: 0.49	0.24	0.0001098	0.03
ALGA0113531	5,901,175	T: 0.55	G: 0.45	-0.22	0.0002927	0.05

All markers are located on SSC6.

**Figure 2:**
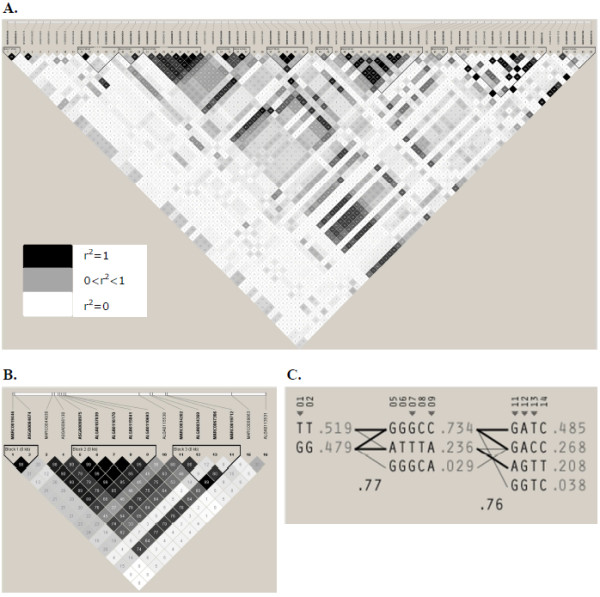
**LD analyses of all 16 significant SNPs (p≤0.05 after FDR), intervening SNPs and haplotypes for the significant SNPs.** The values in the boxes are pair wise SNP correlations (r^2^) and boxes without numbers indicate SNPs in complete LD. The 16 significant SNPs are shown in **B**. Haplotypes for these 16 SNPs are shown in **C**. Each line represents a haplotype and the frequency of the haplotype in this population is given at the end of the line. Haplotypes with a frequency below 2% are not included.

A total of 143 SNPs were located in the region encompassing the initial 6 Mb of SSC6. However, only 16 markers displayed significant associations with skatole in the pig population analyzed and showed three separate regions, two of them defining blocks of consecutive SNPs. In Figure 2A, the linkage disequilibrium (LD) between all the SNPs in the region between 0.64 and 3.9 Mb is shown. Figure 2B shows the LD for the 16 significant SNPs only. The first of these three regions contained two SNPs (MARC0019446 and ASGA0084674) that mapped consecutively to positions 0.63 and 0.65 Mb and show very high LD of r^2 ^= 0.98 (Figure 2B, block 1). The second region spanned approximately 0.4 Mb, from 1.8 to 2.2 Mb, and is the largest block with seven consecutive significant SNP markers. Among all of these SNPs strong LD was detected (Figure 2B, block 2). The third region on SSC6 extended over approximately 0.6 Mb (from 3.3 to 3.9 Mb) and contained five significant SNPs. Unlike the regions discussed previously, there were several non significant SNP markers located between the five significant markers. Furthermore, the SNP MARC0067306 is not in LD with any of the other SNPs in this area (Figure 2B, block 3). This might be due to a wrong position of the SNPs because of a mistake in the build9 assembly. Finally, significant associations were also detected for two isolated markers located at 5.22 Mb (MARC0009863) and 5.9 Mb (ALGA0113531) which are clearly not in LD with any of the other significant SNPs (Figure 2B, SNP 15 and 16).

The means of the untransformed skatole levels for the most significant SNP in each of the regions were very similar across markers, reason why the differences between genotypes were calculated only for the most significant SNP marker in each region (Table 2). Strong differences between genotype classes were identified for the MARC0019446 marker, for which a difference of 39.9 ng/g of skatole in fat between the two homozygotes was detected. In addition, a clear distinction between the homozygous genotypes was also evident for the markers that mapped to the 1.8-2.2 Mb region. The most significant SNP markers in this region were MARC0044930 and ALGA0107039. For the last marker, animals with genotype GG had skatole levels (181.7 ng/g fat) that more than doubled the ones of genotype TT (78.9 ng/g fat). Similar results were obtained for the other SNP markers in the region. The magnitude of this difference of 102,8 ng/g, as well as the intermediate frequency of the unfavourable allele of 23%, may eventually justify the use of this marker in pig selection schemes aiming at reducing the levels of skatole. Finally, the most significant marker in the 3.3-3.9 Mb region (ALGA0034369) also displayed strong differences between the means of the heterozygous genotypes. Animals with genotype GG had skatole levels (165.8 ng/g fat) that greatly exceeded by 89,4 ng/g the ones observed for the individuals with genotype AA (76.4 ng/g fat) (Table 2). These results were similar for the other markers in the region. The unfavourable allele for this marker was found at a frequency of 25% in the population studied, which indicates that the allele is still found at an intermediate frequency and that selection using this SNP marker could possibly be envisioned to reduce skatole levels. The explained phenotypic variance per SNP is given in Table 2. Combining the three most significant SNPs explained 19% of the phenotypic variance (±0.15). The relatively large standard errors of the SNP effects and high LD between the SNPs resulted in a higher explained phenotypic variance for the most significant SNP (ALGA0034369) than combining the SNPs in one analysis. The most favourable haplotype for these three SNPs would be T-G-A in order of location. In Figure 2C, the haplotypes are shown for the SNPs that were defined in blocks using the method of Gabriel and Schaffner [28]. The favourable haplotype is the most common haplotype with a frequency of 0.485. Finally, each of the two isolated SNPs (MARC0009863 and ALGA0113531) explain almost 4% of the phenotypic variance.

**Table 2:** Means and standard deviations of the untransformed skatole levels (ng/g of fat) for selected significant SNP markers on SSC6

SNP	Genotype	N	Mean	Standard Deviation	Explained phenotypic variance
	**TT**	224	74.7	78.0	
**MARC0019446**	**GT**	457	89.1	90.5	0.06 ± 0.06
	**GG**	185	114.6	125.1	
					
	**GG**	507	78.9	85.6	
**ALGA0107039**	**GT**	335	101.8	100.5	0.19 ± 0.16
	**TT**	39	181.7	174.0	
					
	**AA**	496	76.4	79.5	
**ALGA0034369**	**AG**	329	101.6	103.1	0.22 ± 0.18
	**GG**	54	165.8	150.8	
					
	**GG**	208	88.9	109.2	
**MARC0009863**	**AG**	456	86.3	85.6	0.04 ± 0.03
	**AA**	200	103.9	102.3	
					
	**TT**	242	100.2	99.4	
**ALGA0113531**	**GT**	464	85.9	86.7	0.03 ± 0.02
	**GG**	153	93.3	121.2	

The most significant SNP in each of the three clusters identified on SSC6 is listed. The phenotypic variance explained by the SNPs is given with the standard error of the estimate.

The SNPs MARC0019446 and ASGA0084674 are located within intron 1 of the junctophilin 3 gene (*JPH3*). This gene contributes to the stabilization of the junctional membrane complexes and defects in this gene are the cause of Huntington disease-like type 2, a neurodegenerative disorder [29]. However, the way that the *JPH3 *gene could possibly be involved in the regulation of skatole levels in the pig is not clear. A small nucleolar RNA gene, *SNORA70*, also maps in this region. Even though the involvement of non-coding RNA genes in the regulation of complex phenotypes has been demonstrated [30], it is still unclear how this gene could affect porcine skatole levels. A total of four protein coding genes are located in the 1.8-2.2 Mb region on SSC6, however *GINS2 *(GINS complex subunit 2) is the only gene that is characterized so far. This gene is part of a larger complex that plays an essential role in the initiation of DNA replication [31]. The remaining three genes in this region still need to be further characterized. For all genes it is not possible to anticipate the role they play, if any, in the regulation of skatole levels. Based on the build 9 annotation of the pig genome, there are no genes that map to 3.3-3.9 Mb region. The SSC6p region corresponds to the long arm of HSA16 (human chromosome 16), spans approximately 5.4 Mb (from 82.7 to 88.1 Mb) and contains 60 genes. Among the HSA16q genes yet to be annotated in the pig genome are the hydroxysteroid dehydrogenase like 1 (*HSDL1*) and cytochrome c oxidase subunit IV isoform 1 (*COX4I1*) genes. The HSDL1 gene is a member of a family that contains genes that were previously associated with boar taint [32] and is highly expressed in reproductive tissues, including testis [33]. Genes coding for cytochrome oxidases have previously been associated with skatole levels [12-17]. The COX4I1 gene codes for a subunit of a cytochrome oxidase, hence it is possible that it may also be involved in the regulation of porcine skatole metabolism, given its biological role and SSC6 position.

Previous studies had identified several QTL for skatole on SSC6 [9,10], including a QTL for skatole levels (detected by a sensory panel) in the initial part of chromosome 6. This QTL was detected at position 29 cM of the linkage map used in the study, and was flanked by microsatellite marker SW1353 [10]. A search for the SSC6 position of this microsatellite marker revealed that it maps approximately at 7 Mb, a location in the neighbourhood of the significant SNP markers identified in the present study. The resolution of QTL studies using microsatellite markers is lower, when compared with genome-wide association studies comprising thousands of SNP markers, which may cause QTL to be identified at significant distances from their causative genes and/or mutations. Hence, it was not possible to indicate if the QTL identified by Lee et al. [10] was caused by the same genes and/or mutations highlighted in this work.

None of the candidate genes investigated earlier for skatole were detected in the present study. *CYP2A6*, a gene for which associations with elevated levels of skatole in fat had been detected [17] maps to position 33.6 Mb on SSC6, a region that in our study did not reveal any evidence for the presence of markers that were significantly associated with skatole levels. Likewise, the role played by the *CYP2E1 *gene in the regulation of skatole levels has been previously demonstrated [12-16]. This gene maps to SSC14, a chromosome where no significant associations were detected in the present study. Hence, this gene does not seem to be affecting the levels of skatole in the pig population analyzed.

## Conclusion

This study confirmed the increase in resolution of the PorcineSNP60 bead chip in genome-wide association studies compared with traditional linkage studies using microsatellite markers. Several SNP markers located on the distal region of SSC6p were found to be significantly associated with skatole levels. These markers are grouped in three different clusters, altogether located in a region that spans the initial 6 Mb of SSC6. The number of protein-coding genes located in those clusters was low and poorly characterized. However, this study provides evidence for the presence of genes and/or mutations located in the proximal section of SSC6 that affect skatole levels in the pig. The differences between skatole levels for the two homozygous genotypes for several SNP markers were large and single SNPs explained up to 22% of the phenotypic variance. These SNPs may be used in future selection schemes aiming at reducing skatole levels in the pig.

## Authors' contributions

AMR performed statistical analyses, prepared figures and tables and wrote the manuscript. ND participated in sample collection, organization of the genotyping experiment, conducted statistical analyses and contributed to the writing of the manuscript. EFK and JWMM were involved in planning the project and experimental set up. HB participated in the discussion and evaluation of statistical results. RPMAC was involved in DNA collection and the organization of the genotyping experiment. MAMG participated in the analysis and discussion of the data. BH participated in the analysis and discussion of the data, coordinated the study and contributed to the writing of the manuscript. All authors have read and approved the final manuscript.
